# ‘I am proud of how I handled it’. Exploring the impact of the COVID‐19 pandemic and related restrictions on well‐being of adults with severe mental illness using qualitative methods

**DOI:** 10.1111/hex.13983

**Published:** 2024-02-13

**Authors:** L. van Rijn, M. J. Metz, P. R. van der Velden, P. Mathijsen, W. E. Swildens, A. F. A. Schellekens, W. Cahn, M. M. Milota, J. R. Zinkstok

**Affiliations:** ^1^ Department of Psychiatry Radboud University Medical Center, Donders Institute For Brain, Cognition, and Behavior Nijmegen The Netherlands; ^2^ Department of Psychiatry, Brain Center University Medical Center Utrecht Utrecht The Netherlands; ^3^ GGz Breburg Institute for Mental Health Care Breda The Netherlands; ^4^ Tranzo Scientific Center for Care and Wellbeing Tilburg University Tilburg The Netherlands; ^5^ Altrecht, Institute for Mental Health Care Utrecht The Netherlands; ^6^ Inholland University of Applied Science Amsterdam The Netherlands; ^7^ Julius Center for Health Sciences and Primary Care University Medical Center Utrecht Utrecht The Netherlands; ^8^ Karakter Child and Adolescent Mental Health Care Nijmegen The Netherlands

**Keywords:** COVID‐19 pandemic, expert‐by‐experience, mental health, participatory action research, qualitative research, resilience, severe mental illness

## Abstract

**Background:**

The coronavirus disease 2019 (COVID‐19) pandemic and related restrictions globally impacted mental health, particularly for those with pre‐existing severe mental illness (SMI). This qualitative study examined how adults with SMI perceived the effects of the COVID‐19 pandemic and related restrictions in the Netherlands, focusing on their personal recovery, well‐being and daily life, including an exploration of factors influencing these effects.

**Methods:**

Semi‐structured interviews were conducted, audio‐recorded and transcribed verbatim. Reflexive thematic analysis was applied. Purposive sampling was used to ensure diversity of individuals with SMI (i.e., age, gender, diagnosis, cultural background and mental healthcare institution).

**Results:**

Twenty participants (median age: 45 years [SD: 12, 8]; 11 females) were interviewed between May and July 2023. Findings revealed a wide range of experiences: while some individuals reported a negative impact on their existing psychiatric symptoms, others described adaptability, resilience and even positive effects of COVID‐19 restrictions on their mental health and well‐being. Factors influencing the heterogeneic perceptions of the COVID‐19 pandemic and related restrictions include the availability of trusted social relationships and enduring interactions with health professionals.

**Conclusion:**

Personalised support, both socially and professionally, is crucial for addressing fears, building resilience, reducing isolation and encouraging positive coping strategies for individuals with SMI during external crises. In this project, a participatory research approach that integrated the lived experience perspective helped uncover the unique perceptions of people with SMI with regard to the pandemic and related restrictions.

**Patient or Public Contribution:**

The study used a participatory action research approach, with experts‐by‐experience involved in every stage of the project as part of the research team. This included engagement with the funding application process, recruitment strategies for interviews, developing the interview guide, piloting the interview, interpreting findings, and knowledge dissemination activities.

## 1 INTRODUCTION

By February 2024, the global coronavirus disease 2019 (COVID‐19) pandemic caused over 774 million infections and 7 million deaths worldwide.^1^ In addition to the significant impact on physical health, the pandemic also affected mental health.^2^ The rapid spread of the virus, overwhelmed healthcare systems and the absence of a definitive COVID‐19 treatment caused anxiety and concern.^3^ Governments worldwide implemented protective restrictions, such as physical distancing, quarantine and temporary closures (‘lockdowns’), resulting in substantial disruptions to daily life.^4^ Converging evidence indicated a significant increase in self‐reported mental health issues as a result of the pandemic, such as anxiety and depression,^5^, ^6^ in the Netherlands surging 20%–25% higher than prepandemic levels.^7^ According to survey data worldwide, a significant rise in loneliness occurred during the peak pandemic period, characterised by a subjective sense of social isolation.^8^ In addition to these observed effects in the general population, it was hypothesised that individuals with severe mental illnesses (SMI) may potentially experience even more pronounced biological, psychological and socioeconomic consequences due to the pandemic and associated restrictions.^9^, ^10^, ^11^ SMI is defined as a psychiatric disorder necessitating care or treatment, characterised by profound social and societal constraints, which can be both antecedents and consequences of the psychiatric disorder. This condition endures over an extended duration, typically spanning several years, and is not of a transient nature.^12^

Individuals within the group of SMI are most frequently diagnosed with chronic psychotic illness (with or without concurrent substance use disorder); other diagnostic categories include personality disorder and autism spectrum disorder.^13^ It is well known that even before COVID‐19, people with SMI experienced significant disparities in physical health compared to the general population, particularly an increased risk of obesity, asthma, diabetes and stroke.^14^ These health disparities made them particularly vulnerable for COVID‐19 infections,medical complications, hospitalisation and/or prolonged illness duration.^15^, ^16^ Multiple studies substantiated the concerns about the well‐being of people with SMI, indicating that this vulnerable group encountered distinct challenges related to accessing healthcare, managing mental health and sustaining social connections during COVID‐19.^17^, ^18^, ^19^, ^20^, ^21^, ^22^

In contrast to these negative effects of the COVID‐19 pandemic, a recent quantitative review found no clear pattern of change in mental health symptom severity and associated outcomes in adults with pre‐existing mental health conditions.^23^ This systematic review included 37 quantitative studies reporting on one or more of the following outcome measurements: symptom severity, social functioning (assessed through self‐administered questionnaires on social participation and loneliness), quality of life, suicide behaviours and self‐harm in people with pre‐existing mental health conditions. Symptoms of various psychiatric disorders (e.g., depression, anxiety, eating disorders, schizophrenia, bipolar disorder and posttraumatic stress disorder were compared before and during the pandemic, with heterogeneous results. For instance, while depressive symptoms appeared to improve, findings were inconclusive or mixed regarding changes in anxiety and eating disorder symptoms. Additionally, general psychopathology and mental distress did not alter significantly at the onset of the pandemic. Limited evidence was found for additional outcomes including social functioning, quality of life, suicidal behaviour and self‐harm, as only one study addressed these measures either prepandemic or at specific pandemic points.

These contradictory quantitative findings underscore the need for a more in‐depth qualitative analysis in order to generate hypotheses regarding the potential reasons for the divergent results. This study aims to examine how individuals with SMI perceived the effects of the COVID‐19 pandemic and the related restrictions in the Netherlands, with a specific emphasis on their (mental) health, well‐being and the influence on their daily lives. Furthermore, we explored the factors that influenced these effects.

## 2 METHODS

We conducted a qualitative study at two regional mental healthcare institutions in the middle and southern parts of the Netherlands (Altrecht, GGZ Breburg), addressing the impact of COVID‐19 and related restrictions on people with SMI in the Netherlands. We opted for a participatory action research (PAR) design, involving individuals with first‐hand mental health experience.^24^ PAR promotes a collaborative and inclusive research approach, prioritising the perspectives of those directly impacted by the topic. This ultimately enhances the pertinence of the research and adds to a more extensive and holistic comprehension of the topic.^25^, ^26^, ^27^

The primary research team was diverse and multidisciplinary, including an expert with first‐hand experience of mental illness (P. M.), three psychiatrists (J. R. Z., A. F. A. S., W. C.), three senior researchers (W. E. S., M. M. M., M. J. M. of which two specialising in qualitative research (M. M. M., M. J. M.) and two researchers with a medical background and training in qualitative research (L. R., P. R. V.). Monthly meetings were convened by the team to deliberate on various aspects of the study, including the formulation of recruitment strategies and ongoing monitoring of the project's contributions to both academic advancements and community requirements.

### 2.1 Participants and recruitment

Participants were individuals with SMI^12^ (a) aged 18 years and older and (b) who either received care before the onset of the pandemic in February 2020 or sought care during the pandemic for a mental health issue for which they had previously received treatment. Individuals without the ability to give informed consent, and people with insufficient Dutch language skills were not included. We used purposive sampling to ensure diversity of individuals with SMI (i.e., age, gender, diagnosis, cultural background and mental healthcare setting). Potential participants were empowered to self‐assess their mental well‐being and determine their preparedness for the interviews. This approach allowed them the freedom to decide if they felt comfortable and ready to share their experiences related to COVID‐19. All participants had capacity to consent. Our recruitment activities and interviews were conducted simultaneously, affording us the flexibility to assess interviews during the process and adapt our recruitment priorities. After conducting eight interviews, we adjusted our approach to achieve a more balanced representation of men and non‐Western individuals. Additionally, we noted a significant increase in responses from participants affiliated with a specific institution, prompting a shift in our focus to another setting after completing the first ten interviews. Following Braun and Clarke's^28^ reflexive thematic analysis approach, data saturation was not the aim of recruitment and analysis, but the intention was to broadly and meaningfully explore individual patient experiences on the impact of the COVID‐19 pandemic and its restrictions; the size of the data set was also influenced by time constraints and the availability of respondents.

At GGZ Breburg (mental healthcare institution 1), initial contact and recruitment were conducted via healthcare providers, namely, experts‐by‐experience and nurse specialists. At Altrecht (mental healthcare institution 2), recruitment took place during telephone interactions conducted by research assistants (psychologists) as part of the routine outcome monitoring process. Of those who fulfilled the inclusion criteria, expressed interest in participating and agreed to share their personal information, contact details were sent to the investigator. Before giving informed consent, potential participants were contacted by telephone to provide additional information; an information letter was also sent. Interviews were audio‐taped, transcribed verbatim and anonymized.

### 2.2 Expert‐by‐experience involvement

**Figure 1:**
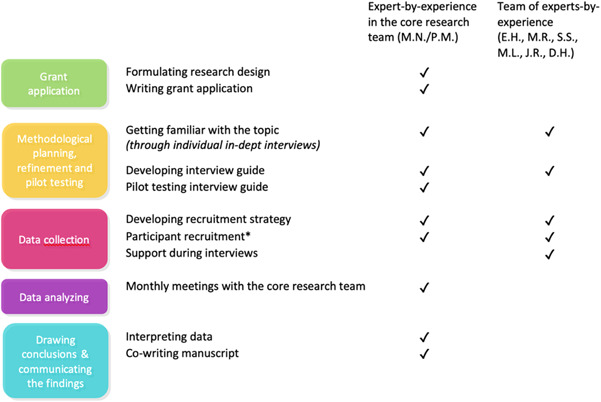
Expert‐by‐experience involvement. *Including weekly telephone meetings to discuss and adjust recruitment strategies.

‘Experts‐by‐experience’ are individuals employed due to their personal first‐hand experience with mental health challenges. They work to improve service quality, combat discrimination, advocate for change and inspire those using mental health services.^29^ Using a PAR approach, the current study engaged an expert‐by‐experience (P. M.) in every stage of the evaluation as part of the primary research team and as coauthor of this study.^27^ Furthermore, an additional team of experts‐by‐experience (E. H., M. R., S. S., M. L., J. R. D.H.) provided insights into the topics essential for discussion during the interviews and assisted in participant recruitment, an example of how the collaboration with experts‐by‐experience pertains to the development of the interview guide. Before interviews, the lead author individually consulted members of the experts‐by‐experience team about the draft topic list, leading to the inclusion of more questions on Dutch COVID‐19 restrictions. The revised list was subjected to pilot testing with the experts‐by‐experience team, and further feedback was obtained from the expert within the core research team. The finalised topic list emerged only after incorporating these feedback rounds. For an overview of expert‐by‐experience involvement, see Figure 1.

### 2.3 Data collection

**Figure 2:**
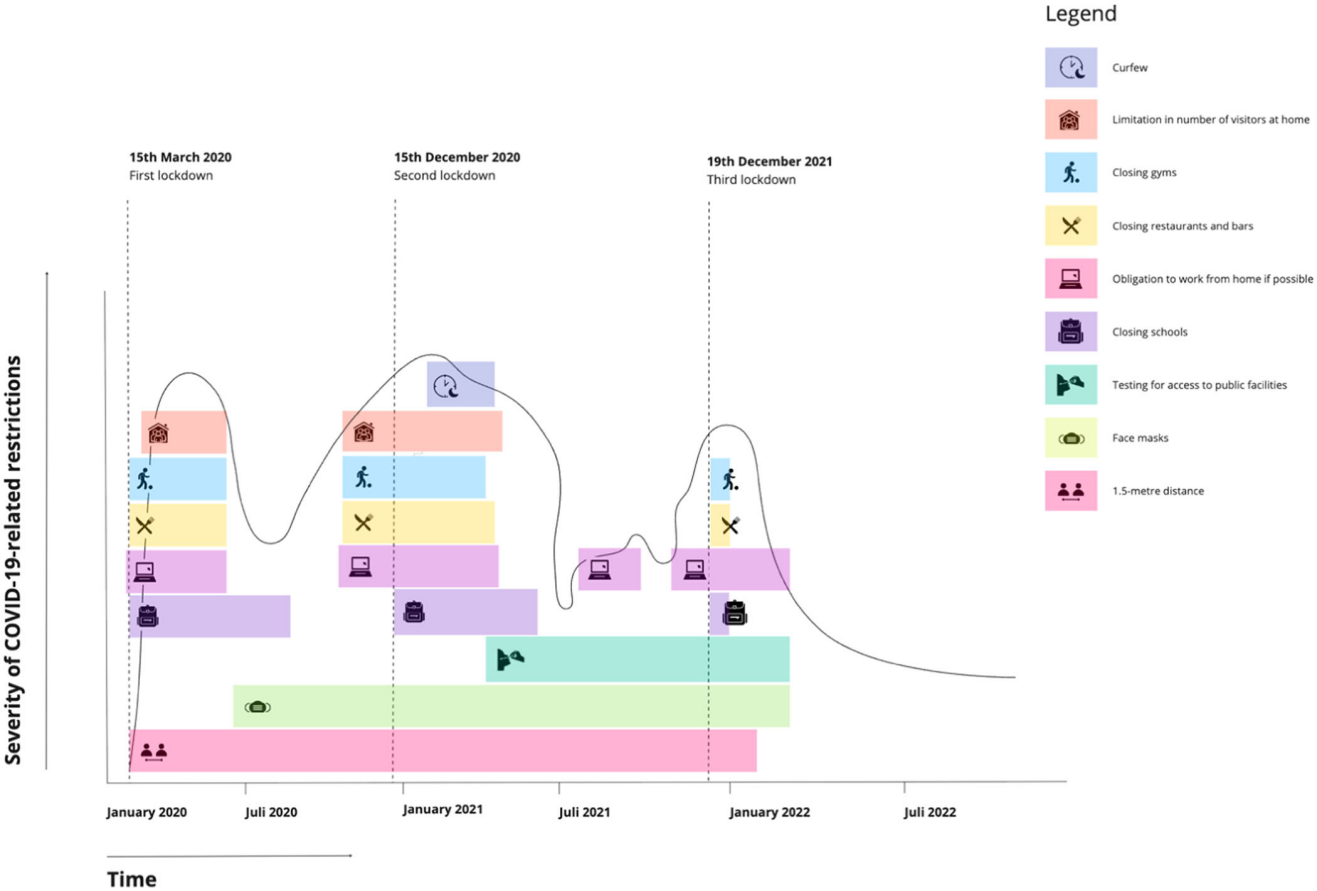
Timeline of COVID‐19‐related restrictions in the Netherlands. COVID‐19, coronavirus disease 2019.

Researchers L. R. and P. R. V. conducted the semi‐structured individual interviews together, with the exception of two interviews, during the period from May to July 2023. During the interviews, one researcher assumed the role of the primary interviewer, while the other participated as an observer. The researchers debriefed after each interview; during these debriefing sessions, the researchers shared and discussed their observations, experiences, reflections and insights obtained from the interaction with the interviewee. The authors did not have any prior (therapeutic) relationships with the interviewees. Interviews were conducted in Dutch face‐to‐face or remotely via Zoom, based on each participant's preference. During the interviews with residents in supported living facilities, an independent expert with lived experience was present to provide support to the interviewee. Participants received a €15 shopping voucher as a token of appreciation for their participation. For the semi‐structured interview guide, see Supporting Information S1: File 1. To refresh participants' memory about the various restrictions and lockdowns, a timeline of the national COVID‐19 restrictions was created and shared with participants before the interview^30^, ^31^ (see Figure 2). Participants were instructed to depict their own experiences using the timeline format, with a peak representing high impact and a trough indicating low impact.

### 2.4 Data analyses

Researchers (L. R., P. R. V., M. M. M.) used reflexive, iterative and thematic analysis to identify themes using ATLAS.ti software.^32^, ^33^, ^34^ The six‐phase systematic approach included familiarisation, coding, generating initial themes, reviewing and refining themes and finalising the thematic structure. Transcripts were first coded deductively, then inductively for additional themes. L. R., P. R. V. and M. M. M. independently read three transcripts, discussed observations and created the initial code tree. L. R. and P. R. V. further refined the code tree with input from other research team members (M. J. M., J. R. Z., P. M.). L. R. coded the remaining transcripts, adjusting codes when necessary. M. M. M. translated interview excerpts into English. The study adhered to the consolidated criteria for reporting qualitative research.^35^

Discussions with experts‐by‐experience within our team highlighted the critical significance and widespread aspiration articulated by individuals coping with SMI to adopt a broad perspective, transcending traditional diagnostic categories. Consequently, we decided to forego detailed diagnostic classifications when structuring the data and conducting the thematic analysis. This methodological choice also acknowledges the diversity in experiences and challenges within this cohort. Our aim was to gain a deeper understanding of how individuals managing severe psychiatric conditions perceive and navigate the extensive repercussions of the pandemic. We recognised that their circumstances are shaped by their condition as well as environmental factors such as social contexts and access to healthcare.

### 2.5 Ethical considerations

The regional Medical Ethics Committee of East Netherlands declared that the Medical Research Involving Human Subjects Act (WMO) did not apply to this study (reference number: 2022‐16087). Participants provided written informed consent before participation in the study.

## 3 RESULTS

**Figure 3:**
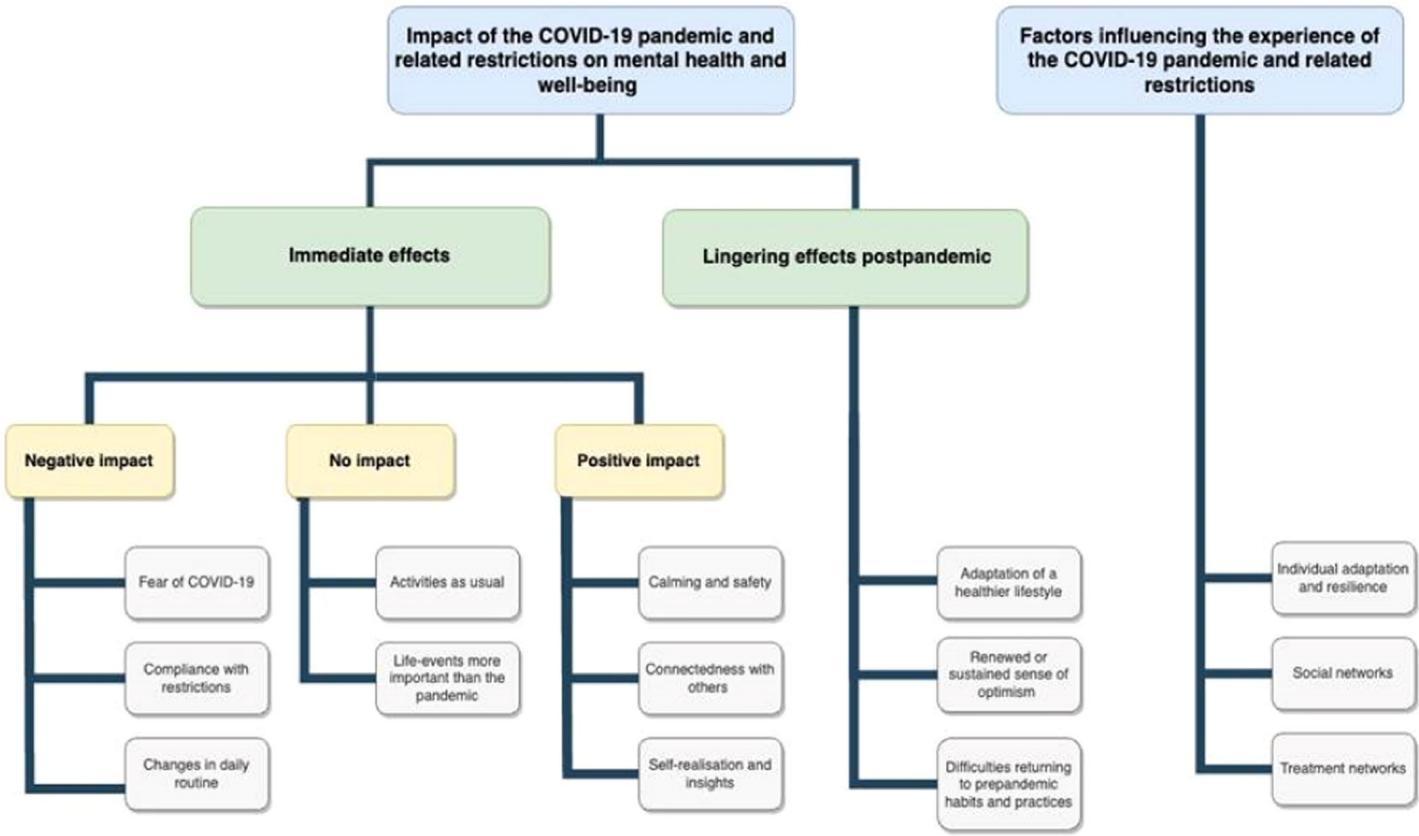
Schematic summarising major themes of the COVID‐19 pandemic and related restrictions. COVID‐19, coronavirus disease 2019.

Between May and July 2023, a total of 20 adults with SMI were enroled and completed the interview, with a mean interview length of 54 min. Participant characteristics are shown in Table 1. The interview guide (see Supporting Information S1: File 1) comprised questions in two categories, and data were grouped accordingly: (1) Impact of the COVID‐19 pandemic and related restrictions on mental health and well‐being and (2) factors influencing the experience of the COVID‐19 pandemic and related restrictions (Figure 3). Thematic analysis resulted in themes and subthemes as, discussed below, illustrated and substantiated by quotes from the data.

**Table 1:** Participant characteristics.

		*N* (%)
Participants (*N*)		20
Gender (F/M/ND)		
Female		10 (50)
Male		9 (45)
Not disclosed		1 (5)
Age (years)		
Mean (SD)		45 (12,8)
Range		27–72
Mental healthcare site		
Mental healthcare institution 1		11 (55)
Mental healthcare institution 2		9 (45)
Living situation		
Alone		10 (50)
With partner, parents or roommates		3 (15)
Supported housing		7 (35)
Highest level of education		
High school or less		7 (35)
Vocational education (Dutch: MBO)		6 (30)
Higher education (including polytechnic and university)		2 (10)
Unknown		5 (25)
Currently employed or studying		5 (25)
Infected with COVID‐19^a^		17 (85)
Self‐disclosed mental illness^b^		
Chronic depression		4 (20)
Enduring psychotic illness including schizophrenia		3 (15)
Schizoaffective disorder		3 (15)
Autism spectrum disorder		3 (15)
Personality disorder		2 (10)
Bipolar disorder		1 (5)
Somatic symptom disorders		1 (5)
Anxiety disorder		1 (5)
Unknown		2 (10)

Abbreviations: COVID‐19, coronavirus disease 2019; F, female; M, male; ND, not disclosed.

^a^ In the period from the beginning of the pandemic to the day of interviewing.

^b^ Participants may self‐disclose >1 mental illness.

### 3.1 Impact of the Covid‐19 pandemic and related restrictions on mental health and well‐being

#### 3.1.1 Immediate effects

Some individuals reported a negative impact of the COVID‐19 pandemic and its restrictions on their mental health and well‐being, while others reported no or positive impact. Furthermore, individuals could have experienced negative aspects on certain themes, while experiencing positive or no impact on other themes. Relevant themes are presented for negative, no and positive impact of COVID‐19 restrictions.

##### Negative impact

###### Fear of COVID‐19

Participants consistently reported heightened anxiety linked to the virus outbreak, notably due to the fear of contracting COVID‐19, particularly among those with compromised physical health. As one participant mentioned,

‘In case people had covid‐like symptoms, I would actually cancel appointments, purely out of fear. If I were to contract it, how would I recover from it?’ (Participant 5).

Notably, a significant proportion of respondents who contracted the virus reported that the actual symptoms were less severe than anticipated. A participant answered the question of what it was like to have COVID‐19 with the following: [speaking of corona] ‘Honestly? Piece of cake. And then I think: did I really stress about that?’ (Participant 5).

However, a minority experienced worsened mental health issues either upon contracting the virus or due to its long‐term somatic consequences, as is illustrated in the following citation:

I experienced ongoing symptoms of anxiety and panic, and I often felt like I couldn't breathe properly or sometimes as if I was suffocating. Then, on top of that, I would experience the impact of the pandemic, and it would worsen even further. (Participant 13)

Some participants experienced heightened stress due to fearing virus transmission to others. Several shared accounts of the virus' significant impact on their loved ones, expressing distress over vulnerable family members. Tragically, some also lost family members or friends to the virus.

###### Compliance with restrictions

The imposed restrictions were perceived as a restriction of freedom by a significant proportion of the participants. Some described this as feeling imprisoned, while others drew comparisons to the Second World War. For example, one participant said, 'It was ‘Like being in prison while sitting at home. Outside you don't feel comfortable, inside you don't feel good. There was no way out’ (Participant 13).

Several individuals recognised the importance of adhering to the restrictions, which led to uncertainty among some about the correct way to comply with them. Doubts about meeting others while experiencing flu‐like symptoms, and uncertainty about the duration of quarantine after infection were common. One individual developed delusional ideas in which he perceived himself as the cause of the increasing transmission rate (*R*‐value) due to the extent to which he adhered to the imposed restrictions. Some expressed cautious disagreement with the implemented restrictions, while others considered them to be completely nonsensical. Consequently, adhering to these restrictions became challenging for them. In one case, this disagreement resulted in friction with mental healthcare professionals (MHCPs), leading to an escalation of tensions and subsequent transfer to a solitary confinement.

Finally, multiple individuals described how the lack of prospects for improvement in the restrictions had an impact on how they felt. According to one:

For me, it felt like I was constantly giving up a piece of freedom, and I think that's what made it mentally very difficult for me. Instead of measures being lifted, new measures kept being added. (Participant 1)

###### Changes in daily routine

For many respondents, the restrictions resulted in the reduction or cessation of contact with friends, colleagues, fellow patients and/or family members, leading to increased social isolation and feelings of loneliness. Several individuals reported experiencing feelings of sadness as a consequence. For some, this loneliness was temporary during quarantine, while for others, it persisted throughout the entire restriction period. One participant described it as follows:

[Before the pandemic,] I already experienced feelings of loneliness. As a result, [during the pandemic] I felt even more alone. And yes, those thoughts came to my mind: nobody cares about me. I don't matter, see, I truly am alone. Yes. Along with that thought also came the belief that things would never get better. (Participant 18)

In addition, the disruption of daily routines led to feelings of boredom among a considerable number of individuals interviewed. As a coping mechanism, some respondents explored new hobbies to alleviate the monotony (see also Theme 4).

Moreover, as a result of this sense of boredom, other participants developed unhealthy lifestyle habits during the pandemic. They reported adopting unhealthy diets, experiencing an increase in smoking, use of alcohol and drugs, change in their sleep routine and spending more time engaging in sedentary activities, such as gaming or watching online series. For example, one participant told us,

I have used drugs before, but mainly during the coronavirus period. Yes, you get bored. And I think there is a specific group that gets bored at home. Then you start listening to music, doing drugs, or you start dating [via internet]. People need something to occupy themselves. (Participant 17).

##### No impact

For a subset of participants, the restricitions had minimal to no impact on various aspects of their lives. Several reasons were identified that help explain this limited impact.

###### Activities as usual

Certain participants reported that their essential activities could continue as usual, allowing for the preservation of their daily routines. These activities included tasks such as grocery shopping and walking, as well as the day‐to‐day pursuits of individuals residing in supported living facilities. Moreover, a subset of participants had limited engagement in various activities before the pandemic, so the restrictions had minimal effect on their overall activity levels.

Well, at that time, I wasn't doing much, so there wasn't much change either. I was already spending a lot of time at home, and not much was happening. I was often alone. (Participant 4)

###### Life‐events more important than the pandemic

[Referring to a period during the COVID‐19 restrictions of low mood after a loved one passed away] My mood did fluctuate, but the COVID‐19 restrictions had absolutely no influence on my mood in that time. (Participant 4)

As illustrated in the quotation above, some participants noted that other life events, unrelated to the pandemic, had a stronger influence on their emotional well‐being. These events demanded more attention, thus reducing the perceived impact of pandemic‐related restrictions. For example, one participant experienced a life‐altering accident, while another grieved the loss of a loved one (unrelated to COVID‐19). These personal experiences shaped their perceptions and responses during the pandemic.

##### Positive impact

Some participants experienced positive effects as a result of the pandemic and its restrictions, which can be attributed to several factors.

###### Calming and safety

Because I also had much more time for myself all at once. I didn't have appointments. A bit of online stuff, okay, but then I didn't have much to do. That gave me space. (Participant 19)

Fewer social activities during the pandemic led to a sense of calm and peace for some participants, offering a welcome break from the demands of social interactions.

In addition, several participants experienced a sense of tranquility due to the decreased presence of people on the streets. This created an environment with fewer stimuli for them.

Well, the first thing that comes to mind is the curfew. I felt a lot better in my living room when there was no one on the streets because I have some psychosis. So, that felt good to me. It's because I'm just a bit paranoid. (Participant 16)

Some felt safe and comfortable precisely because of the restrictions, and mentioned appreciating personal space and reduced contagion risk. Face masks provided anonymity for them, reducing feelings of recognition and judgement based on appearance. As one participant told us:

I found the distance that became the norm very pleasant. I liked it because I didn't have direct contact with people and therefore couldn't be startled or experience anything. But that has more to do with my issues when I don't feel well. (Participant 15)

###### Connectedness with others

The sense of humanity was comforting. We were all human beings. We saw each other. That was very positive. You suddenly became concerned about your therapist if they were not present. (Participant 8)

Some participants experienced a positive impact through a sense of connectedness with others, including peers and healthcare providers, as well as with the broader society. The common denominator was the shared experience of facing the same limitations and challenges imposed by the spread of the virus and the implemented restricitons. Engaging in shared activities, such as following the news together, was mentioned as a communal moment that fostered a positive sense of belonging.

###### Self‐realisations and insights

Participants explored novel insights and values during the COVID‐19 pandemic. Several expressed gratitude for good health and meaningful social connections. Moreover, some participants acknowledged their capacity to exert a positive influence on those around them, emphasising their role in contributing to the well‐being of others.

I am proud of how I handled it. And that it really is the essence of who I want to be and that I am genuinely engaged with my fellow human beings and willing to make sacrifices for them. (…). I think I am just proud that I have discovered myself, in a positive way. (Participant 15)

#### 3.1.2 Lingering effects postpandemic

During the interviews, the restrictions had already been lifted, allowing our participants to retrospectively assess the enduring repercussions of the implemented restrictions. The ensuing discussions encompassed three subthemes: (1) Adoption of a healthier lifestyle; (2) renewed or sustained sense of optimism; and (3) difficulties returning to prepandemic habits and practices.

##### Adoption of a healthier lifestyle

‘We did go for a lot of walks. I had mental health support workers and they would say: “You need to get out. We are going to take a walk”. So, then we would go for a walk together’ (Participant 8).

As illustrated here, a few participants experienced positive lifestyle changes because they explored new activities during the pandemic. Many reported that they walked more outside and for longer, often with friends, family or MHCPs.

Several participants made efforts to maintain healthy lifestyle habits and routines. They recognised the importance of a nutritious diet, sufficient sleep and regular physical activity for their mental well‐being. This awareness motivated them to strive for better adherence to these habits.

I made sure I got enough sleep, I made sure I took my medication, I made sure I took my extra medication. So, I was taking good care of myself, I just made sure that I was just doing everything I can just to keep me on track with my mental health. (Participant 2)

##### Renewed or sustained sense of optimism

While reflecting on the COVID‐19 restrictions, respondents varied in the responses about the emotions that they reported feeling over time; most transitioned from fear to hope and relief, influenced by vaccine availability and declining infection rates, which instilled a sense of optimism. For instance, one participant told us: ‘I do think occasionally about what happened or how things were, but other than that, no. Just moving on with my life’ (Participant 13).

‘But now the sun is starting to shine a bit and things can change after all. Corona is starting to subside and there is light again’ (Participant 13). Relaxation of restrictions allowed resumption of daily lives and a continued recovery journey. Some managed independently, while others acknowledged support from friends, family or MHCP.

##### Difficulties returning to prepandemic habits and practices

However, the easing of COVID‐19‐related restrictions also brought about new challenges for certain individuals. A few participants struggled to reintegrate into social activities due to the prolonged absence from regular social environments and face‐to‐face interactions, making the process challenging. For one participant, going back to school after the prolonged period at home was difficult: ‘When we had to go back to school physically, I started experiencing heart palpitations and I had thoughts like, “What would people think of me if I suddenly show up there?”’ (Participant 2).

After the restrictions were lifted, some participants, particularly those who had experienced anxiety during the pandemic, continued to fear that they would contract COVID‐19. Heightened awareness of virus transmission from the pandemic also contributed to concerns about general health risks during social interactions and in public spaces.

I also think partly because it was strongly emphasized back then, (…) And then I started overthinking in my mind: Oh, so everything I touch now can make me sick. If someone is having a cold, I now avoid them, whereas before it wouldn't have mattered to me. (Participant 18)

### 3.2 Factors influencing the experience of the COVID‐19 pandemic and restrictions

The extent to which the COVID‐19 pandemic and related restrictions impacted individuals with SMI varied. This theme explores the factors that influenced the level of impact and is divided into three subthemes: (1) Individual adaptation and resilience; (2) social networks; and (3) treatment networks.

#### 3.2.1 Individual adaptation and resilience

Despite the challenges posed by the restrictions, the interviews revealed stories and experiences of recovery among the participants. Resilience and adaptability were key elements of this process, as individuals showcased their ability to navigate and adjust to the new circumstances brought about by the pandemic. Participants demonstrated various strategies that contributed to their recovery journeys. First and foremost, participants showed a proactive approach by taking control of their daily activities. They recognised the significance of maintaining their well‐being and showed resilience in the face of their fears. They actively sought ways to fulfil their needs, even when confronted with limitations. One example of this resilient behaviour can be found in the following citation: ‘Getting groceries, going to the shop. You have to find the courage. Still being busy, doing things you have to do in life. I am grateful for that and proud of it’ (Participant 13).

In this regard, a substantial proportion of participants adopted a mature coping strategy in relation to compliance with restrictions. They carefully balanced potential risks against both their own mental well‐being and the well‐being of others. This was done because strict adherence to the measures was not always feasible or aligned with the individual needs of the participants. In certain circumstances, informed decisions were made to occasionally deviate from the restrictions to address specific requirements and safeguard well‐being.

Since omicron b.1.123 started circulating, I began to adhere less and less to the restrictions. Especially because I had the feeling that after almost two years, my mental health was starting to suffer more than I deemed it worth. (Participant 20)

Furthermore, participants showed perseverance, resilience and resourcefulness by finding innovative solutions and alternative methods for self‐care practices. Some continued their regular activities, while others explored new hobbies or pursued indoor activities. ‘I started entertaining myself at home with various crafting activities. I simply enjoyed being creative, and this significantly improved my daily live’ (Participant 1).

#### 3.2.2 Social networks

In addition to individual efforts, participants highlighted the importance of support during restrictions. Religion provided comfort and strength for some. Social support from partners, friends, family, peers, pets and MHCP was crucial for many. Regular communication and interaction through phone calls, video calls and safe‐distance meetings combated isolation and fostered a sense of connection and belonging. For most individuals in supported housing, peer support proved to be of high value. Sharing experiences and actively listening to each other provided a profound sense of understanding and recognition. As one participant mentioned: ‘We were unable to go to the [Name common area], we had to stay home (…) I was glad that [Name peer] came to drink coffee; otherwise, I would have been alone all day’ (Participant 10).

Moreover, several participants mentioned minimal changes in their social interactions due to pre‐existing limited social networks. As a result, their social connections and engagements remained relatively unchanged, leading to a perceived limited impact during the COVID‐19 restrictions. For example: ‘It wasn't too bad. I already lived alone, I already did my groceries alone, so not much changed for me because I was pretty much always alone’ (Participant 14).

#### 3.2.3 Treatment networks

Participants receiving outpatient care frequently reported that their scheduled appointments with healthcare providers proceeded with social distancing or that they transitioned to video calls. Although all respondents perceived video calls as less personal, the majority was deemed acceptable due to their necessity. As one participant stated:

I would prefer to attend in person (…) I simply feel more comfortable during face‐to‐face interactions than during video calls. But if there's no other option and I really have to use video calls, then I would do it. (Participant 13)

Some respondents mentioned that prior relationships with their MHCP had a positive impact on how they experienced care during the pandemic. Those participants indicated that they could rely on the support and guidance of their treatment providers and care teams during the restrictions. As one participant said: ‘Despite it being online, there was still contact. Yes, that helped me. Having someone listening to you, the moments of contact, talking, talking online’ (Participant 19).

Other outpatient care‐receiving responders reported negative effects on mental healthcare, such as perceiving group therapy differently in the online format or limited appointment opportunities. ‘Well, the availability for appointments was also just more limited. (…) While it might have been even more crucial during that period to have more frequent appointments’ (Participant 18).

For individuals in supported housing, when a substantial portion of peer support was impeded by the restrictions, some looked forward to increased social support from the care staff. However, a small number of participants found that these hopes were not fulfilled.

## 4 DISCUSSION

This qualitative study explored the impact of the COVID‐19 pandemic and related restrictions on daily life, well‐being and mental health in individuals with SMI, as well as factors influencing this impact. The findings reveal a wide range of personal perspectives and experiences. Some participants reported adverse effects of the pandemic, including concerns about the COVID‐19 virus, challenges in compliance and significant disruptions to their daily routines. In contrast, other participants indicated little to no impact, and in certain instances, even positive outcomes. These included an elevated perception of tranquility and security, heightened interpersonal connectivity, gaining new perspectives (such as appreciation for health and social relationships) and the adoption of a healthier way of life. Factors influencing these impacts include individual adaptation and resilience, as well as pre‐existing social and treatment networks. As the restrictions began to ease, emotions shifted from fear to hope. Yet, for some, social reintegration proved challenging. This was especially true for those who experienced high levels of anxiety during the restrictions and while ongoing COVID‐19 concerns persisted. The heterogeneity of our findings underscores the substantial role that individual factors play in both the nature and extent of the impact. The diversity in personal circumstances and contexts resulted in a variety of experiences; this, in turn, impedes broader generalisations about the effects of the pandemic on individuals with severe psychiatric problems.

The outcomes of our in‐depth qualitative approach align well with quantitative findings as reviewed by Ahmed et al.^23^; they observed a lack of a clear exacerbation in symptoms of various psychiatric disorders. Participants in our study reported a fairly stable mental health situation, but also a negative impact of the COVID‐19 pandemic and associated restrictions on their overall well‐being. These results are consistent with earlier qualitative studies conducted during the early stages of the pandemic, in which a negative impact was reported, including higher incidence of mental distress and pandemic‐related concerns in a comparable population of individuals with SMI.^19^ Similar themes arose in our study, such as encompassing challenges in coping with day‐to‐day functioning and maintaining social connectedness.^17^, ^18^ Interestingly, our findings regarding the adverse effects of the pandemic show notable parallels with research conducted in the general population.^36^, ^37^ This suggests that the negative impact of the COVID‐19 pandemic was not specific to our target population; rather, people with SMI were not exempt from the effects experienced by the general population. This research aimed to illuminate the consequences of the pandemic restrictions on individuals with SMI, however, the findings in this study underscore a fundamental tension in this healthcare domain. On the one hand, it is imperative to acknowledge the specific challenges associated with a chronic condition. On the other hand, it is essential to avoid stigmatisation and stereotyping by pointing out similarities with the experiences of the majority or the ‘norm’ group.

In contrast to most of the existing qualitative literature on the impact of COVID‐19 on people with SMI, it is crucial to underscore that the negative consequences of COVID‐19 were not predominant in the overall experience of our participants. While the majority of participants described negative consequences, a noteworthy proportion of participants reported also minimal or even positive impact from the COVID‐19 pandemic and restrictions. Despite challenges, these individuals stated that their overall well‐being and recovery processes were unaffected (or even positively affected) by the pandemic. A potential explanation for the discrepancy with earlier qualitative literature on individuals with SMI is the timing of previous studies during the early stages of the pandemic (2020).^17^, ^18^, ^19^, ^20^ During this period, infections and fatalities were increasing and no treatment was available. Our findings of a more mixed pattern of impact are supported by other quantitative research indicating that psychological distress resulting from the COVID‐19 outbreak decreased as the pandemic progressed, both in the general population and among individuals with pre‐existing psychiatric conditions.^38^

In the general population, individuals who experienced relatively more pronounced disruptions and uncertainties in their daily lives, such as parents of young children, working adults and those facing financial difficulties, reported higher levels of emotional distress during the pandemic.^39^ Our target group's pre‐existing living circumstances may have mitigated potential losses. For instance, only 25% of the participants were engaged in work or studies at the time of the interviews, a statistic aligned with the literature on employment among individuals with SMI.^40^, ^41^ Many of the participants were unable to find and/or maintain a job due to their disability, and a considerable number of participants lived alone with a limited social network. Our results mimic findings among older‐aged adults, a group in which certain individuals also faced fewer changes and transitions during the pandemic due to pre‐existing living circumstances.^42^, ^43^

The diverse effects observed within our study population can be attributed to several factors. First, the level of social and community engagement among our participants before the pandemic played a pivotal role in their experience of the pandemic. Results of our study emphasise the vital role of support from social networks and MHCP for individuals with SMI during crises like the COVID‐19 pandemic. Those who received support from friends or family via (video)phone calls or maintaining personal appointments despite restrictions reported minimal loneliness and social isolation during lockdowns. This pattern was consistent with observations made before the COVID‐19 pandemic by people with SMI^39^ as well as the general population during the pandemic.^44^, ^45^ A second factor that partly explains the varied effects is that many of the participants resided in a supported living environment. Participants in these settings notably gained from mutual support among peers, which led to decreased burden amid restrictions. However, during the peak of COVID‐19 restrictions, their burden significantly increased when they were forced into isolated home situations. At that juncture, they anticipated more support from MHCP; in their view, this was not always fulfilled. This finding emphasises the importance of social interaction and network maintenance within these community as a means of mitigating the detrimental effect of isolation on their mental well‐being.

For the majority of participants receiving outpatient care, the assistance provided by MHCP was considered significant. Despite limitations in face‐to‐face contact, existing relationships with caregivers were almost always sustained. This consistency and sustained involvement provided reassurance amidst the pandemic uncertainties. This finding emphasises the significance of continuous and engaged mental healthcare provision, as previously documented in the literature.^46^ A dependable support network comprising of both cohabitants and MHCP appears to be crucial in supporting individuals with SMI during emergencies like a pandemic.

The diversity of responses observed in this study emphasises the imperative for flexible and tailored interventions in mental healthcare, particularly in times of crisis such as the COVID‐19 pandemic. Our findings indicate that individual responses to stress are influenced not solely by underlying disorders but by multiple variables. A comprehensive understanding of mental health necessitates a profound insight into the individual as a whole, encompassing social contexts like family dynamics, living conditions and community relationships. Tailored interventions should consider unique strengths, vulnerabilities and coping strategies.

Based on our findings, we urge policymakers to implement timely and proactive measures during crises such as COVID‐19 to address the diverse needs of this vulnerable group. An example could be the continued facilitation of social interaction among individuals residing in protected living environments. Maintaining or even promoting these interpersonal connections would allow individuals with SMI to retain invaluable social networks. In addition, collaboration with lived‐experience experts and MHCP is indispensable in formulating a flexible and responsive policy frameworks in preparation for future crises. Through dialogue and collaboration, we can create policies that cater to the diverse needs of individuals with SMI, thereby optimising their mental health during crises and beyond.

## 5 STRENGTHS AND LIMITATIONS

The qualitative nature of the study allowed for an in‐depth exploration of the experiences and perspectives of individuals with SMI. A major strength of our study was the active involvement of experts‐by‐experience at every stage. They provided valuable first‐hand insights into the challenges of mental health, which enriched the authenticity of our findings. This inclusive approach made our results more credible and empowered participants to speak openly about their experiences and needs.^25^, ^26^

This study also has several limitations. First, the interviews with the participants took place 2 years after the height of the COVID‐19 pandemic and implementation of the lockdown restrictions. Although a detailed timeline of the pandemic‐related restrictions was provided to facilitate the recollection process, the substantial time gap between the restrictions and the interviews could potentially introduce recall bias. Second, psychiatric conditions like paranoid psychosis or severe depression could have influenced the findings. To reduce bias, methods included an interviewer, observer, asking for specific examples and periodic summaries. Despite efforts to minimise influences from psychiatric conditions, including these perspectives actually enriches the data with additional viewpoints, thus enhancing comprehensiveness. Third, while our study primarily examined the broader impact of COVID‐19 restrictions on individuals with SMI, it is important to acknowledge that certain critical topics, such as suicidality, aggression and sexuality, were not extensively addressed. These themes are complex and sensitive, and may warrant further dedicated research. Finally, while the recruitment methodology and purposeful sampling strategy contributed to a broad spectrum of perspectives, encompassing various genders, psychiatric diagnoses, stages of treatment and geographical regions (both urban and rural areas), our recruitment approach warrants further discussion. It was evident that the vast majority of participants had a Dutch cultural background, which limited the inclusion of adults with SMI from non‐Dutch cultural backgrounds in our research outcomes. In addition, the cohort of participants residing in supported housing was sourced from only two distinct supported housing facilities. Therefore, we recommend that future investigations focus on gaining a deeper understanding of the disparities between outpatient care and supported housing across multiple housing facilities.

## 6 CONCLUSION

This PAR study yielded a wide range of experiences among adults with SMI in relation to the COVID‐19 pandemic and related restrictions. While some individuals reported a negative impact on their existing psychiatric symptoms, others described adaptability, resilience and even positive effects of COVID‐19 restrictions on their mental health and well‐being. The findings emphasise the importance of personalised support and resources for individuals with SMI, addressing fears, fostering resilience, mitigating social isolation and promoting positive coping strategies. Tailored interventions, informed by participatory research and incorporating experts‐by‐lived‐experience, are crucial for addressing the unique needs of individuals with SMI in future crises and recovery efforts, especially within the context of pandemic strategies.

## AUTHOR CONTRIBUTIONS

**M. J. Metz**: Funding acquisition; writing—review and editing; supervision; methodology; conceptualisation. **P. R. van der Velden**: Writing—review and editing; investigation; software. **P. Mathijsen**: Validation; writing—review and editing. **W. E. Swildens**: Funding acquisition; conceptualisation; writing—review and editing. **A. F. A. Schellekens**: Funding acquisition; Writing—review and editing; supervision. **W. Cahn**: Writing—review and editing; funding acquisition; conceptualisation; supervision. **M. M. Milota**: Supervision; conceptualisation; writing—review and editing; methodology; software; formal analysis; investigation. **J. R. Zinkstok**: Supervision; conceptualisation; funding acquisition; writing—review and editing; methodology; data curation; project administration; formal analysis.

## CONFLICT OF INTEREST STATEMENT

The authors declare no conflicts of interest.

## DISCLOSURE

During the initial drafting of this manuscript, the authors uploaded various fragments of the text into ChatGPT to improve readability of the text. After using the tool, the authors further revised and edited these fragments. The authors take full responsibility for the content of this publication.

## Supporting information

Supplementary information.

## Data Availability

The data that support the findings of this study are available on request from the corresponding author. The data are not publicly available due to privacy or ethical restrictions.
