# Supplementary material for: ‘I am proud of how I handled it’. Exploring the impact of the COVID‐19 pandemic and related restrictions on well‐being of adults with severe mental illness using qualitative methods
Source: Health Expect. 2024 Feb 13;27(1):e13983. doi: 10.1111/hex.13983 (PMC10862174; doi:10.1111/hex.13983)
Supplement: Supplementary file 1 — Supplementary information. [file HEX-27-e13983-s001.docx]

**“I am proud of how I handled it.” Exploring the impact of the COVID-19 pandemic and related restrictions on well-being of adults with severe mental illness using participatory action research.**

**Supplementary Materials**

**Supplementary file 1 – Interview guide**

**Glossary**

**COVID-19 restrictions:** Restrictions implemented by the (Dutch) government to prevent the spread of the COVID-19 virus, including quarantine, lockdown (closure of hospitality establishments, gyms, essential and non-essential shops), and maintaining a distance of 1.5 meters.

**Social Network:** A group of individuals with whom the patient maintains social contacts, such as friends, family, and acquaintances.

**Attributing meaning:** The sense or purpose of life or existence

**Mental Health:** The manner in which one relates to oneself and others, and how one copes with the challenges of daily life

**Physical Health:** The state or condition of the body

**Lifestyle:** The distinctive way of life of an individual or group based on stable values and norms. It encompasses elements such as nutrition and dietary patterns, physical activity and sports, well-being and relaxation, substance use, and sleep.

**E-health:** The use of information and communication technology (ICT) to support or enhance health and healthcare.

**Topic-list**

1. **Introduction**

Introduction to researchers, aim of the research, duration, anonymity, recording consent, information about the final product

1. **Orientation to the topic**

Overview of COVID-19, pandemic restrictions

*Example of questions*

- *Can you tell us a about yourself?*
- *Did you have COVID yourself?*
- *What were the most important restrictions that stuck with you?*

1. **Life domains**

Mental health, social network, moral and spiritual beliefs, psychical health

*Example of questions*

- *Could you create a timeline in this figure depicting your experiences regarding your mental health during the COVID-19 measures? Can you explain why you drew the line in this manner?*
- *How did the COVID-19 restrictions affect your social activities (positively/negatively)?*
- *What is important to you in life? Has that changed due to the COVID-19 restrictions? Why has it changed?*
- *What actions/activities/things did you engage in for your physical health before the COVID-19 restrictions? What changed for you in terms of those actions/activities during the COVID-19 restrictions(positively/negatively)? How did you experience this?*

1. **Lifestyle**

Sleep, nutrition and dietary patterns, physical activity and sports, well-being and relaxation, substance use

*Example of questions*

- *What do you think of when hearing the word ‘lifestyle’?*
- *How was your sleep/diet/nutrition and dietary patterns/physical activity and sports/well-being and relaxation/substance use before the pandemic restrictions? Did anything change about this during the pandemic restrictions (positive/negative)? How did you experience this?*

1. **Care provision and treatment relationship**

Contact with treatment team, E-health

*Example of questions*

- *Did you utilize mental health care during the COVID-19 restrictions?* *Was it care you were already receiving, or was it for a new issue?*
- *If the treatment contact was different, in what way was it different than before the COVID-19 restrictions (positive/negative)?*
- *During the COVID-19 restrictions, there has been increased utilization of E-health (treatment via computer). How did you experience this (positively/negatively)?*

1. **Closing**

**Summary of interview goals, reflection on process**

*Example of questions*

- *Is there something we did not discuss, but you would have liked to discuss?*
- *What was it like for you to talk about the pandemic period?*
